# Multicompartmentalized Micellar Structures by Gold Nanoparticles Grafted with Diblock‐Copolymer Ligands

**DOI:** 10.1002/cphc.202400747

**Published:** 2024-10-28

**Authors:** Marina Sebastian, Andreas Fery, Arash Nikoubashman, Christian Rossner

**Affiliations:** ^1^ Institut für Physikalische Chemie und Physik der Polymere Leibniz-Institut für Polymerforschung Dresden e. V. Hohe Straße 6 D-01069 Dresden Germany; ^2^ Faculty of Chemistry and Food Chemistry Technische Universität Dresden Bergstraße 66 D-01062 Dresden Germany.; ^3^ Institut für Theorie der Polymere Leibniz-Institut für Polymerforschung Dresden e. V. Hohe Straße 6 D-01069 Dresden Germany; ^4^ Faculty of Physics Technische Universität Dresden D-01062 Dresden Germany; ^5^ Dresden Center for Intelligent Materials (DCIM) Technische Universität Dresden Hallwachsstraße 3 D-01069 Dresden Germany

**Keywords:** Block copolymers, Colloids, Gold nanoparticles, Multicompartmentalized structures, Surface-pinned micelles

## Abstract

We study the formation of hybrid polymer/inorganic colloidal particles with multicompartmentalized structure, comprising gold nanoparticles grafted with polystyrene‐*block*‐poly(methacrylic acid) (PSt‐*block*‐PMAA) diblock copolymer ligands, through experiments and molecular dynamics simulations. The PMAA blocks segregate into small satellite‐like domains that are separated by the polystyrene spacer from the gold nanoparticle core. Dialysis against water leads to the re‐configuration of the formed structures into unique, kinetically trapped pinned‐micelle‐decorated nanoparticles with internal structure.

## Graphical abstract

The solvency‐induced formation of planet–satellite‐like structures via segregation in block‐copolymers end‐grafted to spherical gold nanoparticles is studied in experiments and molecular dynamics simulations.

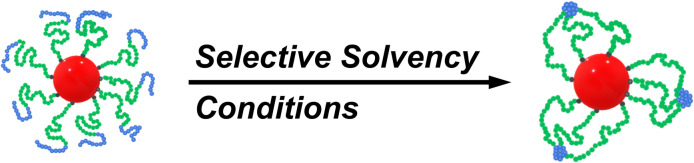

## Introduction

Because of their rich application potential in, e. g., catalysis,^[1]^ autonomous motile systems,^[2]^ or as nanocarriers of drugs,^[3]^ colloidal particles with an internal, hierarchical structure are of abiding interest in soft matter research. Various morphologies of such internally structured colloids have recently been realized. For example, the segregation of ABC‐type triblock terpolymers into multicompartment micelles provides versatile access to “patchy” nanoparticles (NPs), in which either the outer A block or the inner B block can form the core, which is decorated by several discrete B‐ or A‐type domains, respectively (i. e., the “patches”), sterically stabilized by a (solvent‐swollen) C‐type block.[^4^, ^5^] Here, the shape of the core domain can take distinct forms depending on the block ratio,^[5]^ or the core‐forming block can be replaced by an intrinsically phase‐separated inorganic NP.[^6^, ^7^, ^8^, ^9^, ^10^]

**Scheme 1:**
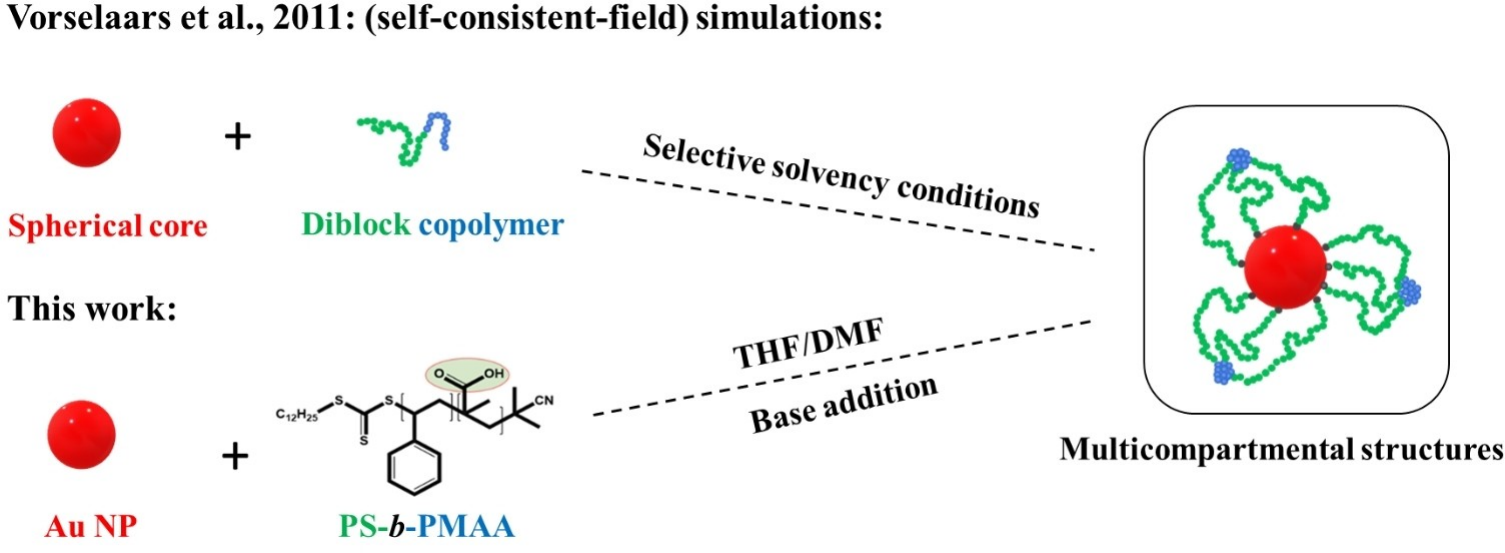
Schematic diagram showing the formation of multicompartmental structures assembled from a spherical core with end‐grafted diblock copolymers and, in particular, Au NPs grafted with PS‐*block*‐PMAA diblock copolymer ligands.

When the individual “patches” are separated from the core domain by a spacer, planet–satellite‐type systems^[11]^ are created. These structures can be accessed with relative ease by linking intrinsically phase‐separated inorganic “planet” and “satellite” particles using appropriate, solvent‐swollen polymer linkers.[^12^, ^13^] However, the formation of planet–satellite‐type structures via segregation in block co‐^[14]^ or ter‐polymers^[15]^ is still challenging. The spatial separation of segregating (A and C) blocks by a central solvophilic (B) block alone may not be sufficient to invoke a spacing between phase‐separated A and C domains, as these domains can still coalesce, supported by looped conformations of the central block.^[16]^ Recently, planet–satellite‐type micellar structures were realized by self‐assembling A_1_B_1_A_2_B_2_‐type tetrablock copolymers in a selective solvent (with solvophobic B blocks and solvophilic A blocks), in which the B_2_ segment forms the micellar core, while B_1_ segments do not merge with this core as long as their volume fraction is small.^[14]^ Comparable structures constituting AB‐type block copolymers grafted onto a solid, spherical NP core have so far only been predicted by self‐consistent field theory calculations (see Scheme 1),^[17]^ but at present there is no experimental counterpart to these predicted structures.

In this contribution, we address this knowledge gap by demonstrating the formation of planet–satellite‐type structures for systems comprising polystyrene‐*block*‐poly(methacrylic acid) (PS‐*block*‐PMAA) diblock copolymer ligands grafted onto (citrate‐reduced) quasi‐spherical gold NPs (diameter ≈ 22 nm). As we will show, structure formation can be initiated by deprotonation of the PMAA block in organic solution. We complement our experiments with particle‐based simulations, which provide molecular‐level insights and allow us to draw relations between the underlying microscopic interaction parameters and the resulting mesoscopic morphologies.

## Materials and Methods

### Chemicals

*Tert*‐butyl methacrylate monomer (TCI), triethylamine (TEA, sigma aldrich), 2‐cyano‐2‐propyl dodecyl trithiocarbonate (sigma aldrich), azobis(isobutynitrile) (AIBN, sigma aldrich), tetrahydrofuran (THF, ACROS organics) and *N,N‐*dimethylformamide (DMF, VWR Chemicals) were purchased at the highest purity available and used as received. Styrene (sigma aldrich) and *tert*‐butyl methacrylate were eluted through a short column of basic activated alumina prior to use. All glassware was cleaned with *aqua regia* and rinsed extensively with Milli‐Q water before use.

### Synthesis of Macro‐RAFT Agent PtBMA

Monomer (*tert*‐butyl methacrylate) (1.8 g, 12.6 mmol), RAFT agent (2‐cyano‐2‐propyl dodecyl trithiocarbonate) (43.6 mg, 0.126 mmol), AIBN (0.012 mg, 7.307×10^−8^ mol) and 25 wt % toluene were degassed with argon for 10 min and then heated to 60 °C for 4 h. The polymeric material was isolated by threefold precipitation into an excess of methanol/water (2/1 v/v).^[18]^ The precipitated polymer was collected and dried *in vacuo* overnight.

### Synthesis of Diblock Copolymer PS‐*block*‐PtBMA

Styrene monomer (2.00 g, 19.0 mmol) and macro‐RAFT agent (P^t^BMA, *vide supra*, 56.0 mg, 10.5×10^−6^ mol) were degassed with argon for 10 min and then heated to 100 °C for 16 h. The polymeric material was isolated by threefold precipitation into an excess of methanol. The precipitated polymer was collected and dried *in vacuo* overnight.

### Hydrolysis of PS‐*block*‐PtBMA^[19]^

Diblock copolymer (0.5 g) was dissolved in 30 mL of trifluoroacetic acid/chloroform mixture (30/70 v/v) and left under stirring overnight at room temperature. Then, all volatiles were evaporated and the solid residue was washed several times with diethyl ether and dried overnight under vacuum at room temperature.

### Synthesis of Gold Nanoparticles^[20]^

A solution of sodium citrate in Milli‐Q water (150 mL, 2.2 mM) was heated with a heating mantle in a 250 mL three‐necked round‐bottom flask for 15 min under vigorous stirring and reflux. After boiling had commenced, HAuCl_4_ solution (1 mL, 25 mM) was injected. The color of the solution changed from yellow to bluish gray and then to soft pink in 10 min. The reaction was cooled down in the same vessel right away following the synthesis of gold seeds until the solution's temperature reached 90 °C. Next, a final NP solution was obtained by sequentially injecting 1 mL of sodium citrate solution (60 mM) and 1 mL of HAuCl_4_ solution (25 mM) and stirring for 30 min.

### Functionalization of Gold Nanoparticles with Block‐Copolymer Ligands

Citrate‐stabilized gold NPs (in water at a concentration of ≈1.4×10^12^ NPs/mL, determined via optical extinction spectroscopy)^[21]^ with an average diameter of ≈22 nm were subjected to ligand exchange to cap their surface with copolymer ligands. Based on the NP concentration and dimension, the polymer functionalization was attempted at target grafting density of 1.0 nm^−2^. The colloidal gold solution (1 ml) was first concentrated by centrifugation (15,600 g, 45 min). Then the supernatant was removed and the NPs re‐dispersed in a minimum amount of the remaining water. The re‐dispersed gold NPs were added into the polymer solutions (1 mL, ≈0.16 mg/mL) in a mixed solvent of DMF/THF 1 : 1 and left undisturbed overnight.

### Deprotonation of the PMAA Block and Staining

To the solution of gold NPs functionalized with block‐copolymer ligands (vide supra), triethylamine (0.1 mL) was added before addition of uranyl acetate (2 %, 20 μL) and incubation overnight. Then, excess polymer and staining agent were removed by centrifugation/re‐dispersion (15,600 g, 90 min, re‐dispersion in THF/DMF).

### Molecular Dynamics (MD) Simulations

The gold NP is modeled as a rigid spherical particle of diameter $a_{NP}=15\;\;nm$ , while the polymers are represented using a bead‐spring model. Each chain consists of 50 monomers, namely one immobile monomer directly grafted to the surface of the NP, 44 PS monomers, and 5 PMAA monomers. All monomers have identical mass m and diameter a=1nm, which roughly matches the length of the corresponding Kuhn segments. The solvent is modeled implicitly, and the solvophobicity of the different components is included in the effective monomer‐monomer pair interactions:

$$U_{mm}\left(\lambda_{ij},r\right)=\left\{\begin{array}{c} U_{LJ}+\left(1-\lambda_{ij}\right)\epsilon ,\;r<2^{1/6}a\\ \lambda_{ij}U_{LJ},\;else \end{array}\right.$$

where $U_{LJ}$ is the standard Lennard‐Jones (LJ) potential with interaction strength $\epsilon =k_{B}T$ and cut‐off radius 3.0a . The dimensionless parameter $0\leq \lambda_{ij}\leq 1$ controls the attraction between a pair of beads of type i and j , and thus their effective solvophobicity. For example, choosing $\lambda_{PS-PS}=0$ leads to purely repulsive, hard‐sphere‐like interactions between PS monomers, and thus good solvent conditions for PS. Polymer bonds are implemented using the finitely extensible non‐linear elastic (FENE) potential:

$$U_{b}\left(r\right)=\left\{\begin{array}{c} -\frac{k}{2}r_{0}^{2}\ln\left[1-{\left(r/r_{0}\right)}^{2}\right],\;r<r_{0}\\ \infty ,\;else \end{array}\right.$$

with spring constant $k=30\epsilon /a^{2}$ and maximum bond extension $r_{0}=1.5a$ . The interactions between the monomers and the central gold NP are modeled using a purely repulsive shifted LJ potential:

$$U_{NP}\left(r\right)=\left\{\begin{array}{c} 4\epsilon \left[{\left(\frac{a}{r-\Delta }\right)}^{12}-{\left(\frac{a}{r-\Delta }\right)}^{6}\right]+\epsilon ,\;r<\Delta +2^{1/6}a\\ 0,\;else \end{array}\right.$$

with $\Delta =(a_{NP}-a)/2$ . We have explored four grafting densities, i. e., $\sigma =0.1\;\;{nm}^{-2},\;0.2\;\;{nm}^{-2},\;0.3\;\;{nm}^{-2},\;and\;0.4\;\;{nm}^{-2}$ , where the grafting points are randomly distributed on the NP surface with a minimum distance of 1nm between them. All simulations have been conducted in the canonical ensemble, where we controlled the temperature using a Langevin thermostat. The equations of motion were integrated using a velocity Verlet algorithm with time step $\Delta t=0.002\tau_{MD}$ , where $\tau_{MD}=\sqrt{ma^{2}/\epsilon }$ is the intrinsic MD unit of time. Initial configurations are created with fully extended chain conformations. Each simulation was run for 10^7^ time steps, and snapshots were saved every 5000 time steps for analysis. We tracked the potential energy of the system as well as the polymer conformations over time to ensure proper equilibration, and discarded the first 500 snapshots from the analysis. All simulations have been performed using the HOOMD‐blue software package (v. 4.7.0).^[22]^

## Results and Discussion

### Functionalization of Gold Nanoparticles with Diblock‐Copolymer Ligands and Formation of Planet–Satellite‐Like Micellar Structures

The diblock copolymers used in this work were synthesized by RAFT polymerization,^[23]^ performed by chain extension of a trithiocarbonate‐type poly(*tert*‐butylmethacrylate) (P^t^BMA) macro‐RAFT precursor with styrene. The molar masses of P^t^BMA and PS‐*block*‐P^t^BMA are 5 and 44 kg/mol, respectively (Table 1). Finally, the block copolymer underwent hydrolysis to produce PS‐*block*‐PMAA.^[19]^ We emphasize that, although trithiocarbonate end groups are sensitive toward, e. g., thermolysis, radicals, and nucleophiles,^[24]^ these functional groups are maintained during the acid‐promoted de‐*tert*‐butylation.^[25]^ A homopolymer of styrene of 44 kg/mol was also synthesized for reference experiments (*vide infra*). Details of the characterization are given in supporting information.

**Table 1:** Properties of block‐copolymers and homopolymers. Apparent number‐ and weight‐average molar masses as well as dispersity values measured by size‐exclusion chromatography.

Polymer	*M*_n_ (g/mol)	*M*_w_ (g/mol)	*Ð*
PS	44,000	47,200	1.07
PtBMA	5300	6600	1.24
PS‐b‐P^t^BMA	44,400	48,500	1.10

**Figure 1:**
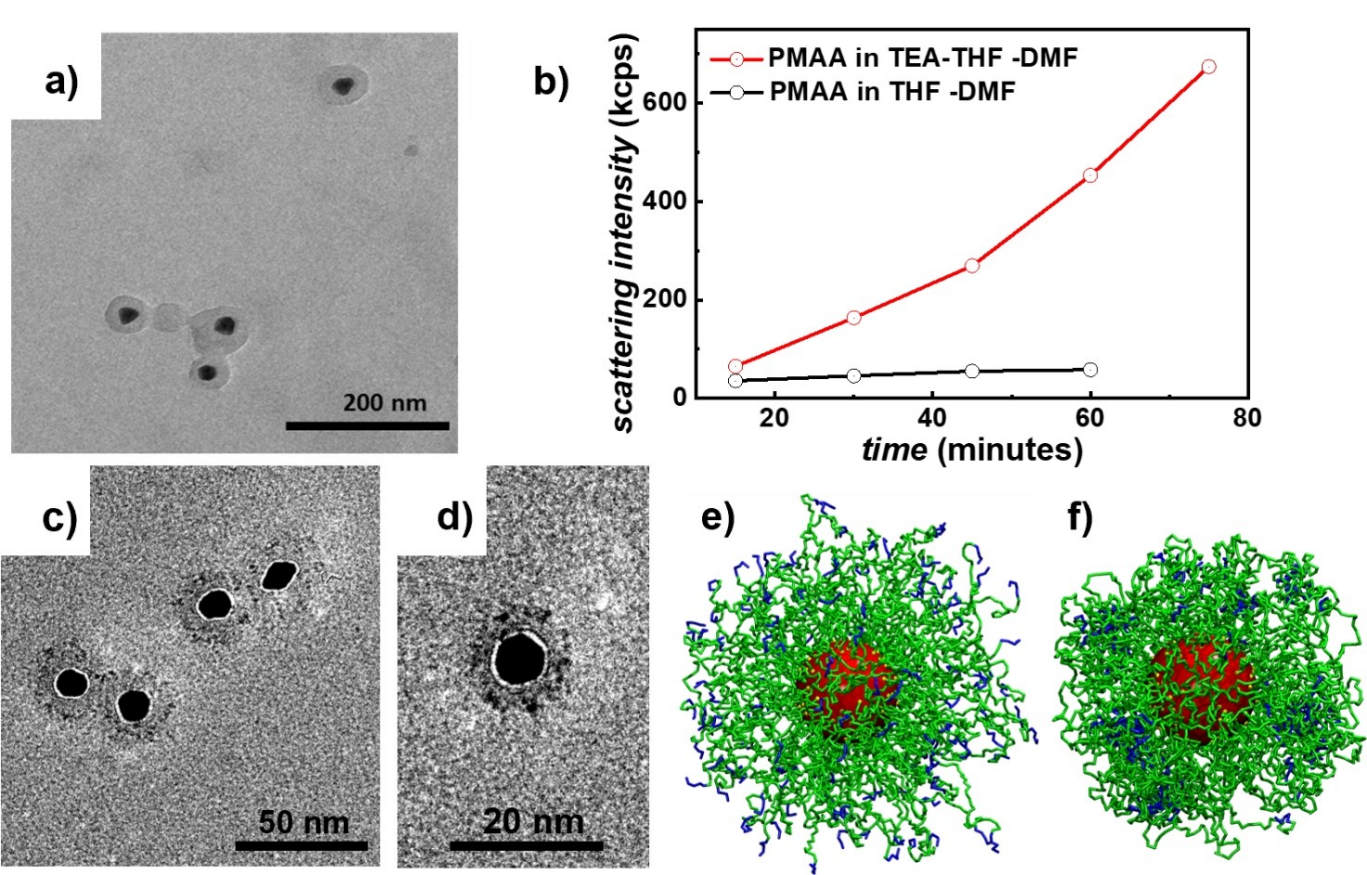
TEM images of gold NPs after functionalization with PS‐*block*‐PMAA and casting from good solvency conditions (a). Scattering intensity of a solution of free PMAA homopolymer in THF/DMF and in THF/DMF after TEA addition (b). Multicompartmental structures formed from Au‐PS‐*block*‐PMAA in THF/DMF/TEA after dying with uranyl acetate (c, d). Simulation snapshot of the block distribution (green: PS, blue: PMAA) at a grafting density of 0.2 nm^−2^, as obtained from MD simulations in a common good solvent for both blocks (interaction parameters: $\lambda_{PS-PS}=0.0,$ $\lambda_{PMAA-PMAA}=0.0,$ and $\lambda_{PS-PMAA}=0.0$ ) (e) and in a selective solvent (interaction parameters: $\lambda_{PS-PS}=0.0,$ $\lambda_{PMAA-PMAA}=1.0,$ and $\lambda_{PS-PMAA}=1.0$ ) (f).

The diblock copolymers were grafted onto citrate‐stabilized gold NPs (22 nm diameter), exploiting the affinity of the trithiocarbonate ω‐termini of the RAFT polymers to gold NPs.[^26^, ^27^] After casting from DMF/THF, i. e., good solubility conditions for both blocks, diblock copolymer‐grafted gold NPs exhibit core‐shell morphologies as indicated by transmission electron microscopy (TEM) (Figure 1a).

In the next step, TEA was added to deprotonate the PMAA block, whereby the solubility conditions for the PMAA block were changed from good to poor. The reduced solubility of pure PMAA homopolymer in THF/DMF after TEA addition is verified by turbidity measurements: Without adding TEA, the PMAA solution exhibited a low scattering count rate; upon TEA addition, the solution turned turbid as a result of starting polymer precipitation (Figure 1b). TEM imaging of these particles after TEA addition and staining with uranyl acetate (UA), a staining agent for polymers with carboxylate groups,^[28]^ revealed the formation of several isolated PMAA domains separated from the gold NP (Figure 1c, d). The gold NP acts as the core around which the entire structure is organized, where smaller spherical domains (i. e., the stained PMAA domains) surround the gold core in a satellite‐like manner. Similar structures are found in our coarse‐grained MD simulations, mirroring these solubility characteristics (Figure 1e, f). These simulations are discussed in more detail below.

The emergence of planet–satellite‐like structures pose the question why the phase‐separated PMAA “satellites” do not adsorb to the gold core to minimize the contact area with the surrounding poor solvent. To investigate whether electrostatic interactions may be involved, we performed electrophoretic (zeta potential) measurements of ungrafted (citrate‐capped) gold NPs and gold NPs grafted with (uncharged) PS ligands. The results (Table 2) show that citrate‐reduced gold NPs grafted with a PS layer retain a negative surface potential in DMF/THF solution, although the absolute value is significantly reduced compared with the initial citrate‐capped NPs in aqueous solution. Thus, remaining surface charges at PS‐layered gold NPs and (partly) deprotonated PMAA domains may give rise to a repulsive interaction between the gold core and the PMAA satellites. However, if such repulsive interactions are critical for the observed structure formation could not be decided based on the performed experiments alone. Thus, to study the effect of brush conformations of the solvent‐swollen PS block independent from potentially contributing electrostatic interactions, we performed MD simulations using a coarse‐grained bead‐spring model (see Materials and Methods section for technical details). For initial screening, NPs with a grafting density of 0.2 nm^−2^ were prepared, which lies within the typically encountered range of such systems.^[13]^ In the simulations, interactions parameters $\lambda_{PS-PS}$ , $\lambda_{PMAA-PMAA}$ , and $\lambda_{PS-PMAA}$ (describing the polymer‐solvent interaction for the PS and PMAA blocks as well as the interaction between the two blocks) were varied systematically, covering purely repulsive, attractive, as well as intermediate conditions. These initial explorative runs reproduced the experimentally observed “satellite” structures for good solvency conditions for the PS block ($\lambda_{PS-PS}=0.0$ ), bad solvency conditions for the PMAA block ($\lambda_{PMAA-PMAA}=1.0$ ) and wetting between the PS and PMAA blocks (for $\lambda_{PS-PMAA}=0.5$ , and $\lambda_{PS-PMAA}=1.0$ ). We emphasize that no additional repulsive interaction between the core and the segregating PMAA block is required to reproduce the experimentally observed “satellite” structures. That is, the conformational degrees of freedom of the solvent‐swollen, NP‐adjacent PS blocks suffice to invoke a spacing between the core particle and the segregated PMAA domains.

**Table 2:** Zeta potential values of gold NPs (Au) and PS‐grafted gold NPs (Au@PS44k) at 25 °C.

Sample	*ζ* (mV)
Au	−57±16
Au@PS_44k_	−19±16

**Figure 2:**
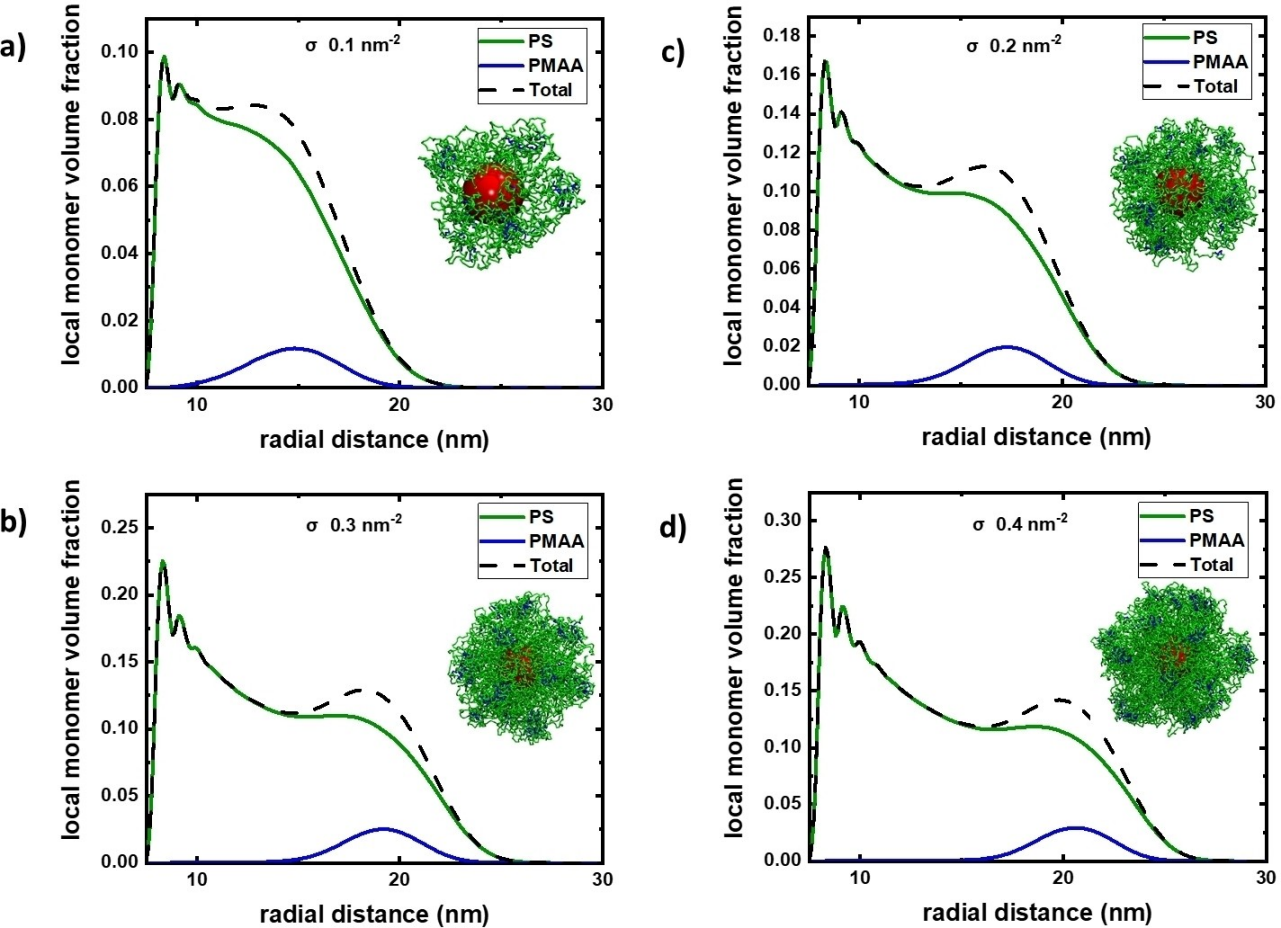
Radial monomer volume fraction for the two distinct blocks PS (in green) and PMAA (in blue) grafted to a solid core, as obtained from MD simulations. The results correspond to interactions parameters: $\lambda_{PS-PS}=0.0$ , $\lambda_{PMAA-PMAA}=1.0$ , and $\lambda_{PS-PMAA}=1.0$ , i. e., good solvency conditions for the PS block and bad solvency conditions for the PMAA block, as well as attraction between PS and PMAA monomers. Grafting densities are: 0.1 nm^−2^ (a); 0.2 nm^−2^ (b); 0.3 nm^−2^ (c); 0.4 nm^−2^ (d).

Next, we investigated the influence of grafting density on structure formation, by varying the grafting density between 0.1 nm^−2^ to 0.4 nm^−2^ in steps of 0.1 nm^−2^ (Figure 2). The formation of “satellite”‐type structures is robust against the variation of grafting density and observed in all studied cases. Further, the MD simulations revealed that the thickness of the PS brush, and thus the distance of the PMAA satellites from the gold NP, increased with increasing grafting density, as expected (see Figure 2 for simulated radial monomer volume fractions). We also observed that a higher number of “satellites” emerge for increased grafting density (Figure S5). In general, the experimentally observed number of formed “satellites” of 10±3 is in reasonable agreement with the simulation results.

### Constrained Surface De‐Wetting and Formation of Internally Structured Pinned Micelles

**Figure 3:**
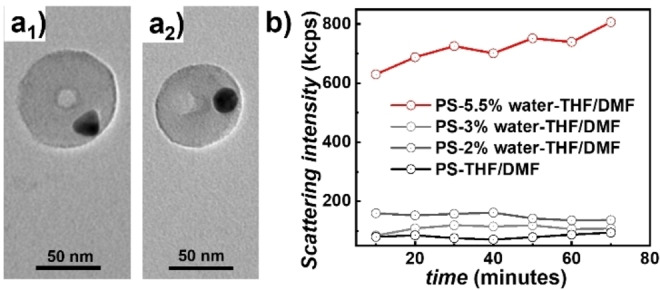
Bright‐field TEM images of surface‐pinned micelle structure with internal, phase separated morphology (a_1_ and a_2_) and scattering of PS in THF/DMF with different water content showing the water content at which PS become insoluble in THF/DMF (b).

PS‐grafted NPs have recently been employed in the formation of so‐called pinned‐micelle structures,[^7^, ^29^] in which unfavorable polymer‐solvent interactions drive the (constrained) de‐wetting of the polymer layer. When block‐copolymers with a more solvophilic outer, NP‐remote block are employed in the de‐wetting, the pinned‐micelle structures can be stabilized by this solvent swollen outer block.[^8^, ^9^] In our hybrid NP system, on the contrary, initially the outer, NP‐remote block is collapsed, while the inner PS‐block remained solvent‐swollen. We were thus intrigued to investigate if de‐wetting of the PS layer can still occur, and what kind of de‐wetted structure would result. Thus, the here functionalized gold NPs in THF/DMF were dialyzed against basic water (pH 8) in a first step, and then against pure water in highly diluted colloidal solutions. Beside NP aggregation, we also observed surface pinned‐micelle structures with an internal, phase‐separated morphology (Figure 3a). Based on the block ratio of the involved PS and PMAA blocks, the spherical domain with the bright contrast in the center of the hybrid NP can be assigned to PMAA, which is engulfed by a PS layer that shows a slightly enhanced contrast. Light scattering experiments of PS in THF/DMF at various water contents were conducted to assess the PS solubility in these mixed solvents (Figure 3b). According to the static light scattering intensity measurements, PS becomes insoluble in THF/DMF at a water content of roughly 5.5 %. This is corroborated by dynamic light scattering measurements that reveal an abrupt increase in hydrodynamic dimension at this water content (Figure S3 and S4). With knowledge of this solvency behavior, we can explain the formation of pinned micelle structures with internal phase‐separated morphology (Figure 3a) as follows: The pinned‐micelle formation is initiated already at comparably low water content during the dialysis process (5.5 % water content). During this process, the initially isolated PMAA domains did merge into one domain, which is eventually kinetically trapped as the PS approaches glassy behavior with increasing water content during the dialysis.

## Conclusions

In conclusion, we demonstrate an experimental realization of unique, planet–satellite‐like structures in diblock‐copolymer‐grafted nanoparticles by segregation of a nanoparticle‐remote block. Comparable structures have so far only been predicted in (self‐consistent field) simulations.^[17]^ In our experimental system, gold nanoparticles, which form an intrinsically phase‐separated core, are grafted with diblock copolymers of styrene (nanoparticle‐adjacent block) and poly(methacrylic acid) (nanoparticle‐remote block). Deprotonation of this PMAA block upon base addition in organic solvent environment renders the PMAA solvophobic, and results in segregated PMAA satellites, separated from the gold‐nanoparticle core by the PS spacer. Using coarse‐grained molecular dynamics simulations, we showed that conformational degrees of freedom of the inner, nanoparticle‐adjacent block prevent the “satellites” from adsorbing to the solid core. Since structure formation is governed by the solubility characteristics of the two blocks, the approach can in principle be transferred to aqueous environments, which provides a wider scope with regard to applications. Turning to poor solvency conditions also for the PS block, hitherto unprecedented surface de‐wetted “patchy” nanoparticles with internal patch structure were observed. We submit that our system may serve as useful template structure, e. g. for nucleating nanoparticle synthesis within the segregated PMAA domains.

## Conflict of Interests

The authors declare no conflict of interest.

1

## Supporting information

As a service to our authors and readers, this journal provides supporting information supplied by the authors. Such materials are peer reviewed and may be re‐organized for online delivery, but are not copy‐edited or typeset. Technical support issues arising from supporting information (other than missing files) should be addressed to the authors.

Supporting Information

## Data Availability

The data that support the findings of this study are available in the supplementary material of this article.
