# Supplementary material for: Multicompartmentalized Micellar Structures by Gold Nanoparticles Grafted with Diblock‐Copolymer Ligands
Source: Chemphyschem. 2024 Oct 28;25(24):e202400747. doi: 10.1002/cphc.202400747 (PMC11648832; doi:10.1002/cphc.202400747)
Supplement: Supplementary file 1 — Supporting Information [file CPHC-25-e202400747-s001.pdf]

ChemPhysChem

Supporting Information

Multicompartmentalized Micellar Structures by Gold Nanoparticles Grafted with Diblock-Copolymer Ligands

Marina Sebastian, Andreas Fery, Arash Nikoubashman,* and Christian Rossner*

Supporting Information

Multicompartmentalized micellar structures by gold nanoparticles grafted with diblock-copolymer ligands

Marina Sebastian,[a,b] Andreas Fery,[a,b] Arash Nikoubashman*,[c,d] and Christian Rossner*[a,b,e]

[a] Marina Sebastian, Prof. Dr. Andreas Fery, Dr. Christian Rossner
Institut für Physikalische Chemie und Physik der Polymere
Leibniz-Institut für Polymerforschung Dresden e.V.
Hohe Straße 6, D-01069 Dresden, Germany.
E-mail: rossner@ipfdd.de

[b] Marina Sebastian, Prof. Dr. Andreas Fery, Dr. Christian Rossner
Faculty of Chemistry and Food Chemistry
Technische Universität Dresden
Bergstraße 66, D-01062 Dresden, Germany.

[c] Prof. Dr. Arash Nikoubashman
Institut für Theorie der Polymere,
Leibniz-Institut für Polymerforschung Dresden e.V.
Hohe Straße 6, D-01069, Germany.
E-mail: anikouba@ipfdd.de

[d] Prof. Dr. Arash Nikoubashman
Faculty of Physics
Technische Universität Dresden,
D-01062 Dresden, Germany;

[e] Dr. Christian Rossner
Dresden Center for Intelligent Materials (DCIM)
Technische Universität Dresden
Hallwachsstraße 3, D-01069 Dresden, Germany

Table of contents

1. Characterization
2. Determination of Polystyrene solubility in THF/DMF/water solvents
3. Formation of surface-pinned micelle structure with internal, phase separated morphology
4. Molecular dynamics simulations
5. References

1. Characterization

NMR spectroscopy

Based on previous work,[S1] the proton signals at 1-1.5 ppm can be attributed to the tert butyl group and proton signals at 1.45–1.95 ppm to the backbone protons of PS. After acid hydrolysis, the proton peak at 1-1.5 ppm disappeared. Therefore, the ^{1}H-NMR spectrum confirms the presence of PMAA and PS segments in the copolymer molecules.

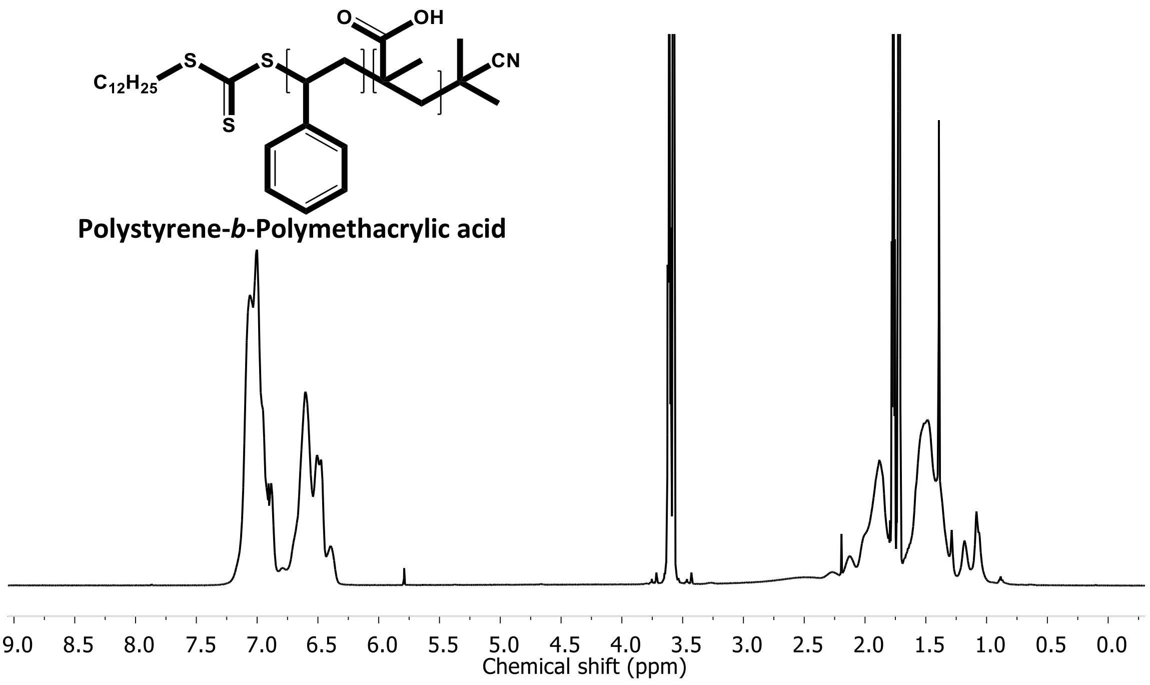

Figure S1: ^{1}H NMR spectrum of the block copolymers used in this work after hydrolysis.

Transmission Electron Microscopy

TEM imaging was performed on a Zeiss Libra-120 TEM instrument at an acceleration voltage of 120 kV, and on a Zeiss Libra-200 at an acceleration voltage of 200 kV. Scattered electrons were blocked with an objective aperture in the focal plane of the objective lens. One drop of the nanoparticle (NP) colloidal solution was applied onto carbon-coated copper grids, and polymer coated gold NPs dyed with Uranyl acetate (UA) were applied on formvar/carbon coated grids and dried in air.

Ultraviolet-visible extinction spectroscopy

UV-vis extinction spectroscopy was performed using a Cary 5000 UV–vis–NIR spectrometer in respective solution using glass cuvettes (optical path length 10mm). Baseline correction was performed by subtracting the spectrum of the pure solvent.

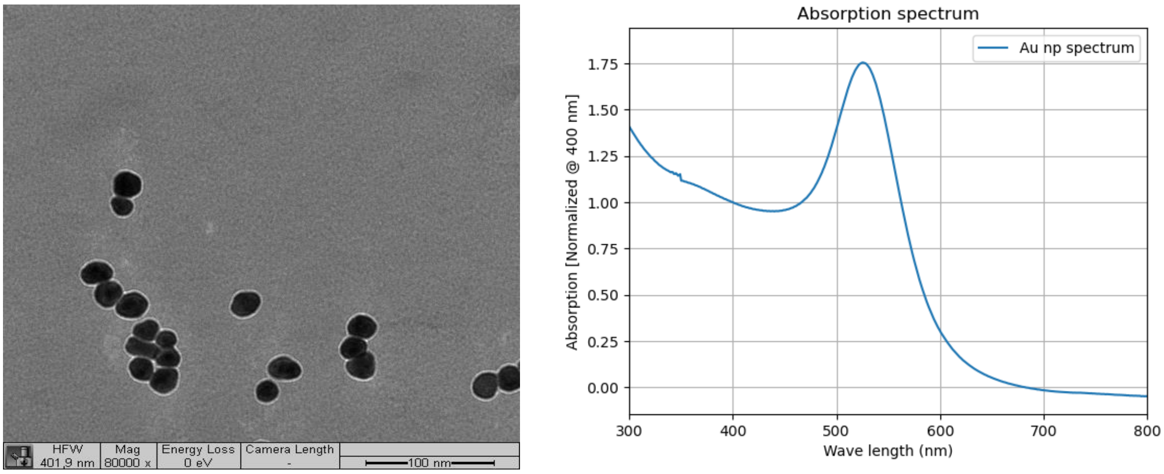

a) b)

Figure S2: TEM image of a gold nanosphere in water drop-cast on a carbon grid (a). Uv-vis extinction spectrum of Au nanospheres in water (b).

2. Determination of Polystyrene (PS) solubility in THF/DMF/water solvents

Solutions of PS (2 mg, 4.5* 10^{-5} mol) in 2 ml of THF-DMF mixtures were taken in separate vials. Different volumes of water were added to it separately, ranging from 0 to 5.5%, and the mixtures were kept undisturbed for 1 hour. Light scattering measurements and hydrodynamic diameter measurement for each sample for overall several minutes was taken.

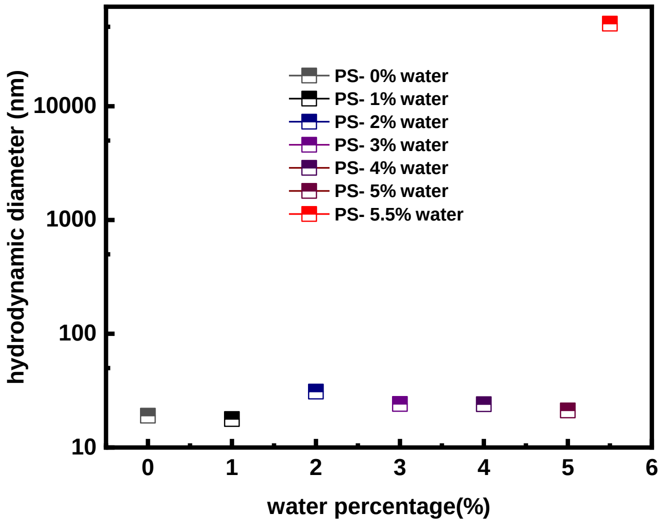

Figure S3: Hydrodynamic diameter data of PS in THF/DMF at different water content showing the water content at which PS become insoluble in THF/DMF.

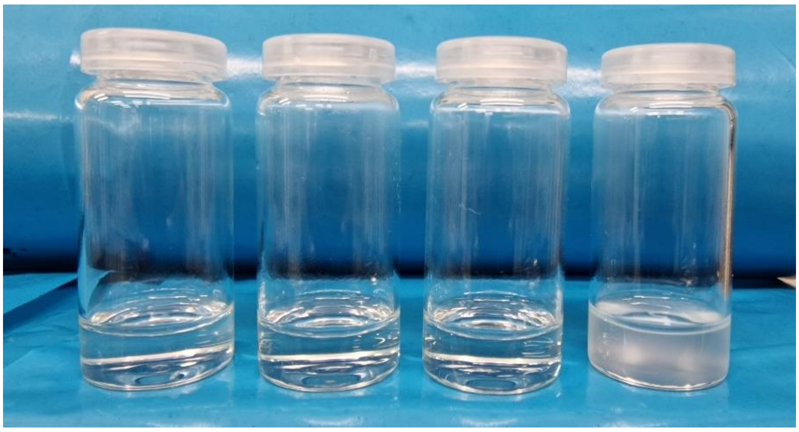

Figure S4: Left-to-right: photographs of 1.0 mg/mL solutions of and PSt50k homopolymers in the THF/DMF at different water percentages (0, 2, 3, 5.5).

With increasing the amount of water content to the above mixture, PS starts precipitating. Specifically, the homopolymer is marginally soluble at a higher water content than 5% and the solution becomes turbid.

3. Formation of surface-pinned micelle structure with internal, phase separated morphology

Using sodium carbonate to adjust pH and MWCO 100 kDa dialysis tubes, gold NPs functionalized with PS-b-PtBMA at a target grafting density of 0.175 nm^{-2} in THF-DMF mixtures were highly diluted with THF-DMF solvent mixture and dialyzed against basic water (pH = 8) for two days. Afterwards, they were dialyzed against pure water (pH 7) for two days. The water in the beaker for above experiments was changed twice in a day. Those particles after dialysis were measured under TEM and UV-Vis spectroscopy.

4. Molecular dynamics simulations

- **Average chains/satellite *Vs* grafting density**

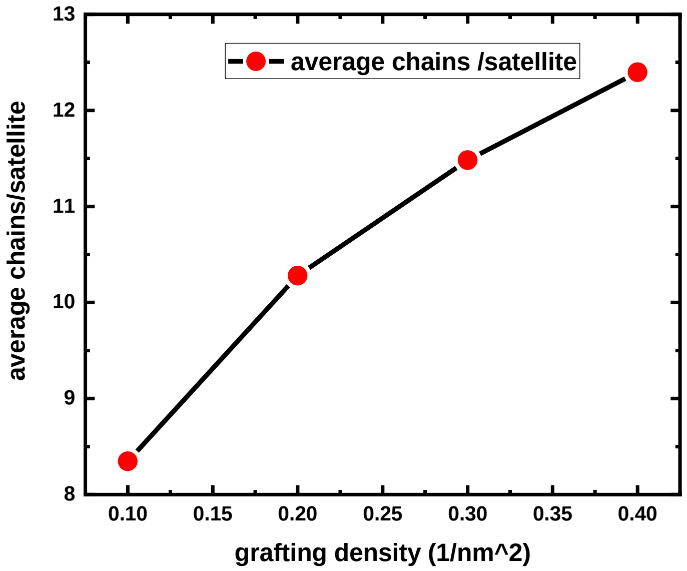

Figure S5: Average number of chains per satellite versus grafting density data of multicompartmental structures showing a higher number of satellites emerge for increase in grafting density at $\lambda_{\text{PS-PS}} = 0.0$, $\lambda_{\text{PMAA-PMAA}} = 1.0$, *and* $\lambda_{\text{PS-PMAA}} = 1.0$.

The influence of grafting density on the multicompartment structure formation where the solvency conditions are good for PS ($\lambda_{PS-PS} = 0.0$), and bad for the PMAA block ($\lambda_{PMAA-PMAA} = 1.0$) and wetting between the PS and PMAA blocks ($\lambda_{PS-PMAA} = 1.0$) is given in the above figure(S5). From these data it is clear that the number of satellites increases with the increase in grafting density, though not proportionally.

5. References

(S1) Yao, D.; Zhang, K.; Chen, Y. Microphase Separation of Poly(Tert-Butyl Methacrylate)-Block-Polystyrene Diblock Copolymers to Form Perforated Lamellae. *Polymer* **2016**, *94*, 1.
